# Cancer incidence following platelet count and C-reactive protein testing across ethnic groups in English primary care: a cohort study of 5 million patients

**DOI:** 10.1136/bmjopen-2026-120128

**Published:** 2026-10-26

**Authors:** Melissa Barlow, Liz Down, Luke T A Mounce, Samuel W D Merriel, Jessica Watson, Tanimola Martins, Sarah E R Bailey

**Affiliations:** 1 Department of Health and Community Sciences, St Luke’s Campus, University of Exeter, Exeter, UK; 2 Centre for Primary Care & Health Services Research, The University of Manchester, Manchester, UK; 3 Centre for Academic Primary Care (CAPC), Population Health Sciences, Bristol Medical School, University of Bristol, Bristol, UK; 4 Department of Family Medicine, Yong Loo Lin School of Medicine, National University of Singapore, Singapore

**Keywords:** Primary Health Care, Early Detection of Cancer, Health Equity

## Abstract

**Abstract:**

**Objectives:**

To investigate whether the association between platelet count or C-reactive protein (CRP) results and 1-year cancer incidence varies by patient ethnicity.

**Design:**

Cohort study using routinely collected health records from the Clinical Practice Research Datalink Aurum, Hospital Episode Statistics Admitted Patient Care and National Cancer Registration and Analysis Service.

**Setting:**

English primary care, with linkage to secondary care records and national cancer registry data.

**Participants:**

Patients aged 40 years or older with a recorded ethnicity, no previous malignancy, and a platelet count or CRP test recorded between 1 January 2010 and 31 December 2017. Ethnicity was categorised as white, Asian, black, other or mixed.

**Primary and secondary outcome measures:**

The primary outcome measures were 1-year cancer incidence following platelet count or CRP testing and variation in the association by ethnicity. Secondary outcome measures were 1-year site-specific cancer incidence and stage at diagnosis.

**Results:**

The study included 4 901 450 patients with a platelet count record and 811 559 with a CRP record. One-year cancer incidence was highest among white patients and lowest among Asian patients. Following a normal platelet count, 1-year cancer incidence was 1.3% (95% CI 1.3 to 1.3%) for white patients and 0.63% (0.60 to 0.66%) for Asian patients; following thrombocytosis, incidence increased to 3.9% (3.8 to 4.0%) and 1.8% (1.5 to 2.1%), respectively. After a normal CRP result, cancer incidence was 1.5% (1.4 to 1.5%) for white patients and 0.81% (0.73 to 0.90%) for Asian patients, rising to 3.6% (3.5 to 3.7%) and 1.9% (1.7 to 2.2%) for a high CRP result, respectively. No significant interactions were found between ethnicity, blood test result and overall cancer diagnosis, and similar ORs were observed across all ethnic groups. However, for colorectal cancer, black patients with abnormal results showed higher ORs compared with white patients, relative to a normal result. The OR for thrombocytosis was 11.1 (7.8 to 15.6) for black patients versus 5.7 (5.4 to 6.0) for white patients (interaction p value<0.001), and for raised CRP was 4.1 (2.6 to 6.6) for black patients versus 2.5 (2.3 to 2.7) for white patients (interaction p value=0.043).

**Conclusions:**

This large primary care study provides reassurance that thrombocytosis and raised CRP appear to have broadly consistent associations with 1-year cancer incidence across ethnic groups in UK primary care.

**STRENGTHS AND LIMITATIONS OF THIS STUDY**

- A key strength of this study was that it addressed an important and unanswered clinical issue and provides general practitioners with more information about the significance of abnormal platelet and C-reactive protein (CRP) test results.
- This study included a large cohort: just under five million patients who underwent a full blood count in primary care and over 800 000 with a CRP record. Such scale provided substantial statistical power to detect differences between groups and, importantly, provided reassurance where no evidence of a difference was observed.
- The use of large, routinely collected primary care data linked to the national cancer registry provided nationally representative evidence of blood test results and subsequent cancer incidence across ethnic groups.
- This study did not account for differences in access to primary care between different ethnic groups, which may contribute to differences in blood testing and subsequent cancer diagnosis.
- Categorising patients by broad ethnic group may oversimplify the complex interplay of cultural, religious, social and demographic factors within and between ethnic groups and may mask important heterogeneity that could influence primary care engagement and subsequent blood testing.

## Introduction

 The National Cancer Plan for England aims to prioritise earlier diagnosis, as well as taking action to reduce inequalities in cancer care.^1^ Inequalities in cancer diagnosis are consistently reported for Asian and black communities in the UK.^2–5^ Despite generally experiencing lower incidence,^6^ patients from Asian and black ethnic groups are more likely to be diagnosed at an advanced stage for some cancer types,^2^ experience longer time to diagnosis^3^ and report worse experiences of cancer care than patients from white ethnic backgrounds.^4 5^ The factors driving these inequalities, particularly the role of primary care in the diagnostic pathways, are not fully understood.

Primary care remains the principal entry point for most cancer diagnostic journeys in the UK and substantial research has focused on improving early diagnosis through this setting.^7^ As cancers develop, they can induce systemic inflammation and alter haematological and inflammatory markers, sometimes before symptoms arise, creating opportunities for earlier detection through routinely requested blood tests. For example, thrombocytosis (platelet count ≥400×10^9^/L) has a positive predictive value for undiagnosed cancer of 6% in women and 11% in men,^8–11^ and English national guidance recommends primary care clinicians to consider lung, colorectal, oesophageal-gastric or uterine cancer in a patient with thrombocytosis.^12^ Platelet count is known to rise in response to infections and inflammation,^13^ and C-reactive protein (CRP), another inflammatory marker, has a positive predictive value of >3% in the detection of cancer in primary care.^14^

However, studies of platelet count, CRP and cancer risk have not examined the potential influence of ethnicity on these associations, despite evidence suggesting variation in their distribution across ethnic groups.^8 9 11 14^ A recent systematic review reported that black patients from a healthy or general population in the USA were more likely to have higher platelet counts and higher CRP levels than their white or Asian counterparts. In contrast, studies in the UK and elsewhere reported no meaningful differences in platelet count by ethnicity, and no UK studies have yet described CRP distributions by ethnic group. Furthermore, it remains unclear whether these blood tests perform equally well in predicting cancer incidence across ethnic groups.^15^

Evidence from studies of specific cancer markers (prostate-specific antigen and cancer antigen-125) has shown variation in both baseline differences in test values by ethnicity in individuals without cancer^16–19^ and variation in test performance for cancer detection in primary care.^20 21^ The reasons for these differences are complex and not fully understood.

This study aims to (1) describe the distribution of platelet count and CRP levels for patients from different ethnic groups presenting to primary care in England, (2) assess cancer incidence following a blood test showing platelet count and CRP levels for patients from different ethnic groups in English primary care and (3) analyse the association of blood test result and cancer stage at diagnosis for different ethnicities.

## Methods

### Data sources

This cohort study used electronic English primary care records from the Clinical Practice Research Datalink (CPRD) Aurum dataset,^22^ linked to the Hospital Episode Statistics Admitted Patient Care (HES APC) dataset^23^ and the National Cancer Registration and Analysis Service (NCRAS) cancer registry.^24^

### Patient selection criteria

Patients were included if they had an ethnicity record in either the CPRD or HES APC datasets, no history of malignancy (other than non-melanoma skin cancer), a platelet or CRP record between 1 January 2010 and 31 December 2017, and were aged 40 years or over at the time of that test. Cohort entry (the index date) was defined as the date of the first eligible platelet or CRP test during the study period, from which follow-up commenced. Separate analyses were conducted for platelet count and CRP level. Accordingly, two cohorts were assembled: one based on the first eligible platelet test and one based on the first eligible CRP test. Patients with both tests could contribute to both cohorts, with cohort entry defined independently for each analysis.

### Outcome variables

Cancer diagnoses were identified from the cancer registry using International Classification of Diseases, Tenth Edition(ICD-10) codes (online supplemental materials 1), and patients were classified as being diagnosed with cancer if a cancer diagnosis was recorded in the year following the date of their blood test. Patients with no cancer recorded in this timeframe (1 year) were classified as not having been diagnosed with cancer, in line with previous work.^8 11 14 25^ The 1-year timeframe was chosen to capture cancers that were present at the time of the blood being tested and to capture the pre-diagnostic rise in haematological indices before cancer diagnosis.^26 27^ Cancer stage was determined using the tumour, nodes and metastasis (TNM) classification. Patients with a T stage of 3 or 4 and/or M stage of 1 were categorised as advanced stage.

### Exposure variables

Ethnicity was categorised into white, Asian, black, other and mixed in accordance with previous literature^20 21^ and the 2021 UK census.^28^ Ethnicity was primarily obtained from CPRD and extracted from HES APC when missing from CPRD, following a previously published algorithm.^21^ A patient’s first blood test result in the study period was selected from the CPRD dataset; platelet count and CRP results were extracted from these records. Included platelet levels were within the range of 50–999×10^9^/L of blood and included CRP levels were between 0 and 500 mg/L.

A high platelet count (thrombocytosis) was defined as a platelet count ≥400×10^9^/L blood in accordance with previous literature,^8 11^ and a high CRP level was defined as ≥7 mg/L, calculated by determining the mean of the upper limit of normal from the laboratories in this study, as described previously.^14^

### Statistical analysis

Multi-level logistic regression models, clustering patients within general practices, were constructed to explore the association between a high test result and diagnosis of cancer within 1 year of test, stratified by test (platelet count or CRP). Differences in this association by ethnic group were explored by including an interaction term. The marginal distributions of the models were used to obtain adjusted cancer incidence estimates by group. Additional models assessed these effects in relation to the 1-year incidence of cancers of specific sites identified from previous findings^11^ and the National Institute of Health and Care Excellence (NICE) guidelines.^8^, including colorectal, lung, oesophageal-gastric and uterine.^8^

Covariates included age group in 10-year bands (40–49 years, 50–59 years, 60–69 years, 70–79 years and 80 years or above), sex (male or female, for cancers that are not sex-specific), smoking status (ever or never), tertile of the Cambridge Multimorbidity Score (CMS) ‘general outcome weighting’ (including an extra category for ‘no identified conditions’),^29^ quintile of the area-based Index of Multiple Deprivation 2015 (IMD)^30^ and body mass index (BMI) (≤18.49 kg/m^2^, 18.5–24.99 kg/m^2^, 25.0–29.99 kg/m^2^, 30.0–39.99 kg/m^2^, ≥40 kg/m^2^, not recorded).

Smoking status, BMI and multimorbidity were all ascertained from records available prior to cohort entry (the index test date). BMI was defined using the most recent recorded measurement before the index date. Patients without a recorded smoking status were classified as never smokers, while those without recorded conditions contributing to the CMS were assigned a score of 0 ‘no identified conditions’. Patients without a recorded BMI were retained in the analysis as a separate ‘BMI not recorded’ category. Multiple imputation was not undertaken because the absence of a BMI measurement in routinely collected primary care data is unlikely to be missing at random.

As different ethnic groups have higher incidences of certain cancer sites, the secondary analysis of stage at diagnosis also included an additional categorical covariate for cancer site.

### Sample size calculations

A sample size calculation determined that a sample size of 1118 per group was sufficient to detect a cancer incidence of 3% with a margin of error of <1 percentage point.

### Patient and public involvement and engagement

This study was developed in consultation with an existing Patient and Public Involvement and Engagement (PPIE) group, as part of a larger programme of research. A dedicated group of three PPIE representatives were recruited for this study, comprising both men and women, and ensuring representation from each of the main three ethnic groups analysed (white, Asian and black), and assisted in the interpretation of results.

### Reporting of results

Analyses were conducted using Stata MP V.18.0. Graphs were produced using Stata MP V.18.5 and GraphPad Prism V.10.2.3 for Windows. Results were reported in accordance with The Reporting of Studies Collected Using Observational Routinely-Collected Health Data statement,^31^ as an extension of Strengthening and Reporting of Observational Studies in Epidemiology statement^32^ (online supplemental materials 2).

## Results

### Patient selection process

Data were received from the CPRD for 5 796 384 patients who had a platelet count record, and 1 509 952 patients with a CRP record. After exclusions based on patient age, date of test, implausible blood test values or units of measure, there were 4 901 450 eligible patients with an acceptable platelet count record, and 811 559 eligible patients with an acceptable CRP record (figure 1). Almost all the patients in the CRP cohort had a recorded platelet count and so also contributed to the platelet cohort (804 086, 99%).

**Figure 1:**
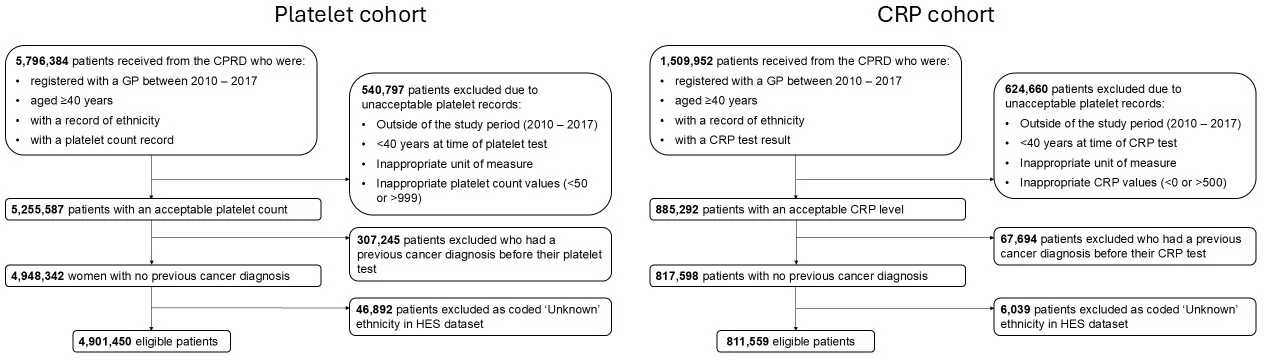
The cohort selection criteria to derive the platelet and CRP cohorts. CPRD, Clinical Practice Research Datalink; CRP, C-reactive protein; GP, general practice; HES, Hospital Episode Statistics.

### Cohort characteristics

The distribution of patients across ethnic groups was broadly similar between the platelet and CRP cohort, with ≤1% of patients assigned to the other or mixed ethnic groups (table 1). Results for patients from the other and mixed groups are detailed throughout the online supplemental materials.

**Table 1:** Cohort characteristic

Ethnicity	n (%)	Sex, % male	Age, median (IQR)	Highest morbidity burden tertile*, %	Overweight or obese BMI, %	Most deprived quintile, %
Platelet count						
White	4 303 178 (87.8)	45.3	58.8 (48.2–70.7)	22.7	69.7	15.7
Asian	312 185 (6.4)	47.4	50.3 (42.9–60.6)	16.2	65.6	25.7
Black	192 444 (3.9)	44.4	49.4 (43.7–58.4)	12.8	79.0	44.0
Other	45 277 (<1)	43.1	49.0 (43.3–57.7)	12.0	70.6	28.2
Mixed	48 366 (<1)	47.8	49.3 (43.2–59.0)	10.8	71.2	28.3
CRP cohort						
White	706 580 (87.1)	41.8	61.0 (49.9–73.3)	26.3	67.9	16.1
Asian	51 755 (6.4)	42.4	54.3 (45.7–64.9)	22.0	65.5	19.1
Black	37 140 (4.6)	38.6	52.2 (46.0–63.0)	18.3	78.5	43.3
Other	8119 (1.0)	38.8	51.0 (45.0–60.8)	16.1	70.0	27.7
Mixed	7965 (<1.0)	43.1	51.3 (44.8–61.3)	13.7	72.2	27.4

* As measured by the Cambridge Multimorbidity Score. Overweight or obese BMI includes patients with a BMI of ≥25 kg/m2.

BMI, body mass index; CRP, C-reactive protein.

There was a slightly higher proportion of females in each ethnicity cohort. The white ethnic group were the oldest with a median age of 59 years in the platelet cohort and 61 years in the CRP cohort. This compared with a median age of 49 to 50 years and 51 to 54 years, respectively. Just under half of all black patients in both cohorts were assigned into the most deprived IMD quintile (43-44%), compared with only 16% of white patients. Black patients were also more likely to be overweight or obese (79%), compared with 66%–72% of the remaining population.

### Distribution of platelet count in patients presenting to primary care by ethnicity

The proportion of patients with thrombocytosis generally increased with age (figure 2a), with the exception of premenopausal women (aged 40–49 years) who had slightly higher levels of thrombocytosis and higher median platelet levels than postmenopausal women, for all ethnic groups (online supplemental materials 3 and 4). For patients aged 50 years or above, the black cohort had lower proportions of patients with thrombocytosis compared with white and Asian patients in the same age groups. A similar trend was seen across the ethnic groups for the distribution of median platelet count values (online supplemental materials 4). A higher proportion of younger Asian patients (40–49 years) had thrombocytosis (4.5%, 95% CI 4.4 to 4.6%) compared with younger patients from other ethnic groups, whereas a higher proportion of older white patients (80 years or above) had thrombocytosis (6.0%, 95% CI 5.9 to 6.0%). Women had higher proportions of thrombocytosis and higher median platelet count values compared with men, regardless of age and ethnicity.

**Figure 2:**
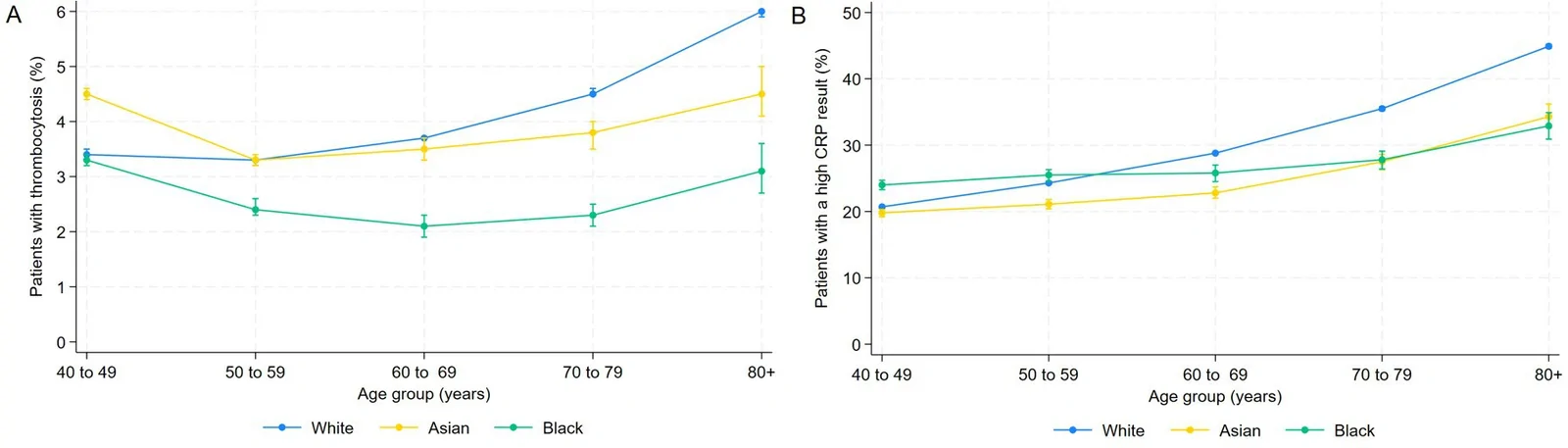
The percentage of patients with thrombocytosis (**A**), and a high CRP result (**B**), stratified by ethnicity and age group. Bars represent 95% CIs. CRP, C-reactive protein.

### Distribution of C-reactive protein level in patients presenting to primary care by ethnicity

The proportion of patients with a high CRP result increased with age, without a premenopausal spike for women as observed with thrombocytosis (figure 2b, online supplemental materials 5). Black patients experienced a more gradual increase in with age than their white or Asian counterparts, which was reflected in a more constant distribution of median CRP values. White and Asian patients experienced a more pronounced rise with age (online supplemental materials 6). There was little evidence of sex differences of median CRP levels or proportion of patients with a high CRP result (online supplemental materials 5 and 6).

### One-year cancer incidence

A total of 91 585 (1.9%) patients were diagnosed with cancer in the year following their platelet count test, and 22 089 (2.7%) in the year after their CRP test. Online supplemental materials 7 presents cancer site and stage at diagnosis. Adjusted 1-year cancer incidence was highest for white patients, and lowest among Asian patients (figure 3). The overall 1-year cancer incidence for white and black patients with thrombocytosis or a high CRP result exceeded 3%, but was under 2% for Asian patients. There were no significant interactions between ethnicity, blood test result and an incident cancer diagnosis, with similar ORs observed across all ethnic groups, including other and mixed online supplemental materials 8 and 9 .

**Figure 3:**
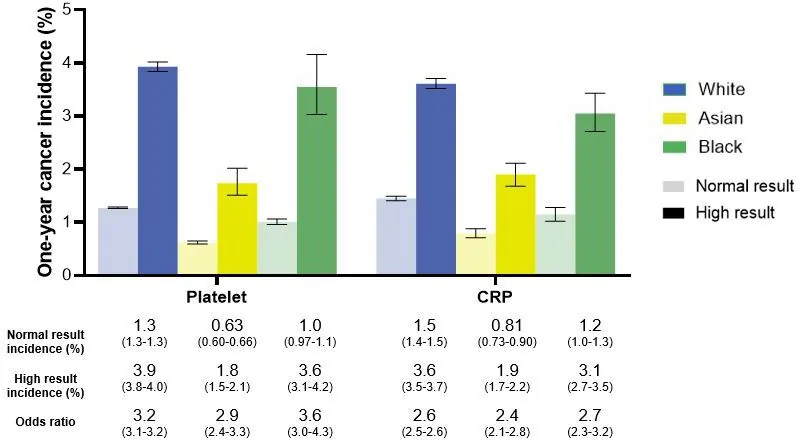
One-year cancer incidence by platelet count and CRP result, and ethnicity. Lighter bars represent a normal test result, and darker bars represent a high test result. Blue bars represent the average 1-year cancer incidence for white patients, yellow bars for Asian patients and green bars for black patients. Black error bars represent 95% CIs. The table reports the incidence (%) and the OR of a 1-year cancer diagnosis with 95% CIs in brackets underneath its corresponding ethnic group and blood marker from the chart above. Adjusted for gender, age group in 10-year bands (40–49 years, 50–59 years, 60–69 years, 70–79 years and 80+ years), sex (male or female), smoking status (ever or never), CMS grouping, BMI category, IMD quintile and year of test. BMI, body mass index; CMS, Cambridge Multimorbidity Score; CRP, C-reactive protein; IMD, Index of Multiple Deprivation.

### One-year colorectal, lung, oesophageal-gastric and uterine cancer incidence

Across colorectal, lung, oesophageal-gastric and uterine cancers, thrombocytosis and a high CRP result were associated with higher 1-year incidence compared with normal results (figure 4). Exceptions were observed for Asian and black women for uterine cancer by thrombocytosis status, and Asian patients for oesophageal-gastric cancer and black women for uterine cancer by CRP status, where there was little evidence of increased cancer risk following a high test result.

**Figure 4:**
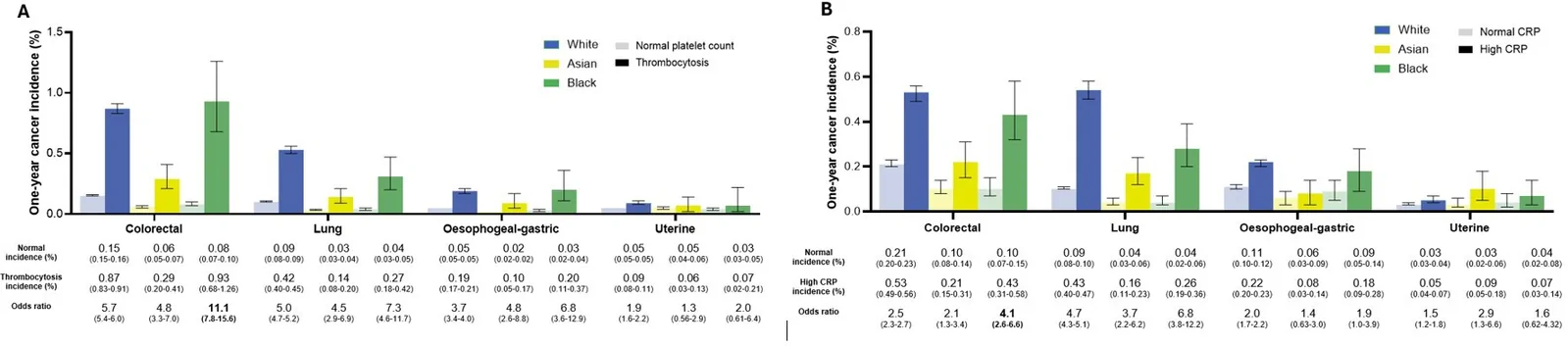
One-year colorectal, lung, oesophageal-gastric and uterine cancer by platelet test result (**A**)/CRP result (**B**), and ethnicity. Lighter bars represent a normal test result, and darker bars represent a high result. Blue bars represent the average 1-year cancer incidence for white patients, yellow bars for Asian patients and green bars for black patients. Black error bars represent 95% CIs. The table reports the incidence (%) and the OR of a 1-year cancer diagnosis with 95% CIs in brackets underneath its corresponding site and ethnic group from the chart above. The uterine cohorts included female patients only. Adjusted for gender, age group in 10-year bands (40–49 years, 50–59 years, 60–69 years, 70–79 years and 80+ years), sex (male or female)*, smoking status (ever or never), CMS grouping, BMI category, IMD quintile and year of test. *Sex was not included as a covariate for the ovarian cancer analyses. BMI, body mass index; CMS, Cambridge Multimorbidity Score; CRP, C-reactive protein; IMD, Index of Multiple Deprivation.

Significant interaction effects were observed between ethnicity and blood test results for 1-year colorectal cancer incidence (interaction p value<0.001 for thrombocytosis and 0.043 for a high CRP result). Among black patients, thrombocytosis was more associated with incident colorectal cancer (OR 11.1, 95% CI 7.8 to 15.6%) compared with white patients (OR 5.7, 95% CI 5.4 to 6.0, p*<*0.001), relative to a normal platelet count. A similar, although weaker, pattern was observed for raised CRP levels, with higher odds among black patients (OR 4.1, 95% CI 2.6 to 6.6) compared with white patients (OR 2.5%, 95% CI 2.3 to 2.7%, p=0.043) (figure 4).

Patients from mixed ethnic groups with thrombocytosis, relative to normal platelet levels, had higher odds of incident oesophageal-gastric cancer compared with white patients (OR 9.0, 95% CI 3.9 to 20.6 for mixed; OR 3.7, 95% CI 3.4 to 4.0 for white; p=0.037). Similarly, women from other groups with thrombocytosis had higher odds of incident uterine cancer compared with white women (OR 13.7, 95% CI 3.3 to 57.3 for other; OR 1.9, 95% CI 1.6 to 2.2 for white; p=0.007). However, with only eight women from the other ethnic group receiving a uterine cancer diagnosis, this result should be interpreted with caution (online supplemental materials 8). Unadjusted 1-year cancer incidence is detailed in online supplemental materials 10.

### One-year cancer incidence of other cancer sites

There was little evidence of ethnic differences in the interaction between ethnicity and blood test result and incident diagnoses of other cancer sites (online supplemental materials 8 and 9). Notable exceptions included kidney cancer and leukaemia. For these cancers, thrombocytosis was more strongly associated with incident diagnosis, relative to a normal platelet count, among black patients than among white patients (OR 10.4, interaction p value=0.046 for kidney cancer and OR 5.78, interaction p value=0.004 for leukaemia). Some evidence of a similar pattern was observed among patients from mixed ethnic groups for leukaemia, though this finding should be interpreted with caution given the small number of leukaemia diagnoses in this group (n=10) (online supplemental materials 8).

### Advanced stage incidence

There was no evidence of differences in the proportion advanced stage cancers in the cohort of patients diagnosed with cancer in the year following their blood test by ethnic group (figure 5). For both blood tests and for all ethnicities, a high result was more indicative of an advanced stage at diagnosis; however, the evidence of this was weak for Asian patients with a high CRP result (OR 1.6, 95% CI 0.9 to 2.3). There was no evidence of differences for the other and mixed groups (online supplemental materials 8 and 9).

**Figure 5:**
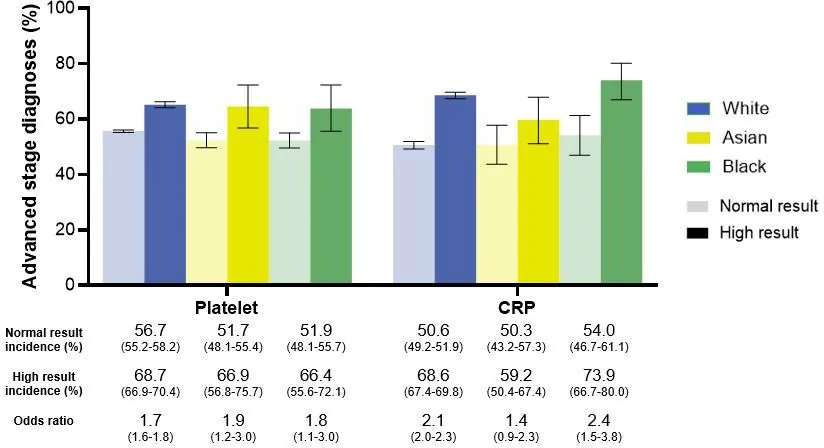
The proportion of cancer patients who were diagnosed at an advanced stage by platelet count and CRP result (normal or high) and ethnicity. Lighter bars represent a normal test result, and darker bars represent a high result. Blue bars represent the average proportion of white cancer patients diagnosed at an advanced stage, yellow bars represent the same for Asian patients and green bars for black patients. Black error bars represent 95% CIs. The table reports advanced stage diagnoses as a percentage (%) and the OR of an advanced stage diagnosis for cancer patients is provided underneath its corresponding ethnic group and blood marker from the chart above, with 95% CIs. Adjusted for gender, age group in 10-year bands (40–49 years, 50–59 years, 60–69 years, 70–79 years and 80+ years), sex (male or female), smoking status (ever or never), CMS grouping, BMI category, IMD quintile and year of test. BMI, body mass index; CMS, Cambridge Multimorbidity Score; CRP, C-reactive protein; IMD, Index of Multiple Deprivation.

## Discussion

This large UK primary care study found the association between thrombocytosis or a raised CRP result for 1-year cancer incidence was generally consistent across ethnic groups and that these blood tests can continue to be used in cancer diagnostic pathways across ethnically diverse populations.

The overarching strength of this study was the very large size of the cohort: just under five million patients who underwent a full blood count in primary care and over 800 000 with a CRP record. Such scale provides substantial statistical power but also carries the potential limitation of increased risk of incidental findings. There was little evidence of ethnic variation in the ability of thrombocytosis or a raised CRP result to detect 1-year cancer incidence, which was largely consistent across each cancer site. There were three notable exceptions for thrombocytosis: colorectal, renal and leukaemia. For these cancers, the OR associated with thrombocytosis, relative to a normal platelet count, was significantly higher for black individuals compared with their white counterparts (OR 11.1, p<0.001 for colorectal cancer; OR 10.4, p=0.046 for kidney cancer; and OR 5.78, p=0.004 for leukaemia). These findings must be interpreted cautiously, given the very large sample size and number of statistical comparisons performed, which increase the likelihood of chance associations. Nonetheless, the well-established links between thrombocytosis and both colorectal and renal cancers suggest that these differences could have genuine clinical relevance.^9 11^ In contrast, the limited evidence for an association between thrombocytosis and leukaemia warrants particular caution, and further work is needed to establish whether this represents a true biological signal, or a statistical artefact.

The relatively higher proportion of patients presenting with a raised CRP level (≥20%, figure 2) compared with those with thrombocytosis (≥2%, figure 1) likely reflects primary care testing practices. CRP testing is often undertaken in the context of suspected inflammation or infection, often supporting clinical decision making regarding antibiotic prescribing. In contrast, platelet count is measured as part of a full blood count, which is requested across a broader range of clinical contexts, with thrombocytosis often occurring as an incidental finding. However, the reasons underlying the differences by ethnicity are less clear. The median platelet counts presented in online supplemental materials 4 indicated that black patients have lower counts than their white and Asian counterparts across all age groups, raising an important concern regarding the use of universal thrombocytosis thresholds in cancer risk estimation. If baseline platelet distributions differ by ethnicity, a similar absolute increase in platelet count may not result in the same likelihood of exceeding current thrombocytosis thresholds, potentially affecting who is identified for further investigation in primary care.

The potential for ethnicity-specific interpretation of any biomarker raises important ethical considerations, particularly in relation to ethnicity-based adjustments to diagnostic thresholds.^33^ Such approaches can oversimplify the complex interplay of socioeconomic, cultural and structural determinants of health and may inadvertently exacerbate health disparities rather than reduce them.^33^ Engagement with PPIE representatives highlighted that patient willingness and ability to engage with primary care services may differ substantially across ethnic groups, influenced by ethnicity-specific cultures and beliefs, religious practices and socioeconomic status. Differences may also arise in whether and when blood tests are offered and accepted. These considerations are supported by a large body of qualitative research.^34–42^ A more responsible approach to evaluating diagnostic tools is to test and validate them in populations that are representative of those in which the tests are intended to be used. In the UK context, this means including ethnically diverse samples that reflect the nation’s multi-ethnic population, and any observed differences by ethnicity should be carefully investigated for their underlying causes, rather than assuming ethnicity alone is the determinant of risk.

The CPRD dataset delivered and analysed in this chapter is largely representative of the population of England and Wales in terms of ethnicity. According to the 2011 UK census (which is most relevant to the study period), 86.0% of the population were white, 7.5% were Asian, 3.3% were black, 2.2% were mixed and 1% were from the other ethnic group. The respective proportions represented in this chapter’s cohort were 87.8% white, 6.4% Asian, 3.9% black, <1% mixed and <1% other. The close alignment with national demographics, together with the large sample size, supports the generalisability of the findings revealed in this study. Importantly, it provides reassurance that the lack of observed ethnic differences in the predictive value of thrombocytosis for cancer incidence supports its use as a universally applicable marker in primary care.

Whether these results are applicable to countries outside the UK is unclear. A recent systematic review found that black individuals in the USA had higher platelet counts than their white counterparts,^15^ a direct contrast to the results found in this study. Different healthcare systems, access to healthcare and cultures may contribute to these differences, and caution should be exercised in generalising the findings from this UK-based cohort to other international populations.

There are several limitations that should be considered. First, differences within ethnic groups, such as the differences between the black African and Caribbean ethnic groups, or the East and South Asian ethnic groups, were not explored. The decision was made to align with common UK practices in ethnicity coding and to prioritise sample sizes, though the significance of heterogeneity within ethnic groups was recently highlighted by findings from the COVID-19 pandemic.^43^ Second, the use of area-based deprivation scores, such as the IMD, does not adequately account for variations of deprivations within areas, which have been shown to significantly differ within neighbourhoods by ethnicity.^44^ Third, it was not possible to determine the clinical rationale for ordering blood tests, whether patients from different ethnic groups present and report to primary care with equal frequency or whether patients were offered and accepted blood tests equally. Fourth, emerging evidence from our research group suggests that relying solely on a patient’s first blood test may not fully capture a patient’s cancer risk. Using alternative approaches, such as random or non-initial tests, may provide more accurate estimates. Nevertheless, this consideration does not affect the overall conclusions or clinical interpretation. Finally, much of NICE’s thrombocytosis guidance was defined in its 2015 update, meaning the majority of data in the present study were recorded before this guidance could have influenced clinical practice.

Overall, these findings provide reassurance that thrombocytosis and raised CRP appear to have broadly consistent associations with cancer risk across ethnic groups in UK primary care.

## Supplementary material

10.1136/bmjopen-2026-120128 online supplemental file 1

10.1136/bmjopen-2026-120128 online supplemental file 2

10.1136/bmjopen-2026-120128 online supplemental file 3

10.1136/bmjopen-2026-120128 online supplemental file 4

10.1136/bmjopen-2026-120128 online supplemental file 5

10.1136/bmjopen-2026-120128 online supplemental file 6

10.1136/bmjopen-2026-120128 online supplemental file 7

10.1136/bmjopen-2026-120128 online supplemental file 8

10.1136/bmjopen-2026-120128 online supplemental file 9

10.1136/bmjopen-2026-120128 online supplemental file 10

## Data Availability

Data may be obtained from a third party and are not publicly available.
