# Supplementary material for: Cancer incidence following platelet count and C-reactive protein testing across ethnic groups in English primary care: a cohort study of 5 million patients
Source: BMJ Open. 2026 Oct 26;16(10):e120128. doi: 10.1136/bmjopen-2026-120128 (PMC13711558; doi:10.1136/bmjopen-2026-120128)
Supplement: Supplementary file 1 [file bmjopen-2026-120128.reviewer_comments.pdf]

PEER REVIEW HISTORY

BMJ Open publishes all reviews undertaken for accepted manuscripts. Reviewers are asked to complete a checklist review form and are provided with free text boxes to elaborate on their assessment. These free text comments are reproduced below.

ARTICLE DETAILS

Title (Provisional)

Cancer incidence following platelet count and C-reactive protein testing across ethnic groups in English primary care: a cohort study of 5 million patients

Authors

Barlow, Melissa; Down, Liz; Mounce, Luke T A; Merriel, Samuel W D; Watson, Jessica; Martins, Tanimola; Bailey, Sarah E R

VERSION 1 - REVIEW

**Reviewer**	**1**
**Name**	**Herold, Zoltan**
**Affiliation**	**Semmelweis University, Department of Clinical Oncology**
**Date**	**09-Apr-2026**
**COI**	**none**

Authors of the article invsestigated cancer incidence and its relationship to platelet and CRP measurements using a large database. The following issues were found.

1. It is suggested to include some extra information in the Introduction. Specifically it was not detailed how platelet and CRP changes with age. There are a few articles about this topic. Moreover, it is worth to mention how cancer develops, specifically the timeframe, why is it interesting to investigate it in this situation. Furthermore, maybe the Authors can mention some of previous works that tried to investigate how platelet and the varios calculated biomarkers (such as the "personalized indicator thrombocytosis", PLR, SII, PIV, etc.) are associated with cancer incidence.

2. In Methods, some additinal details should be included, such as how the various cancer types were recorded in the database (ICD10 codes) and how Authors used these?

3. In most sources the development time of malignant tumors is usually between a 3-5 years range, depending on the source. Authors should include some citation, or give a bit more details on why they chose only to investigate cancers developing within a year timeframw following labor sampling.

4. In the general cohort statitistics it is advised to add an additional (supplementary) table to give details on the cancer details, including the number of cancers developed in total, in the various cancer types (crc, breast, etc.), TNM stages, etc. If these daata were previously published, a citation would also work.

5. In the case of CRP data, it is a bit strange that the percentage of patients with high CRP is between 20-40%. This suggests that the sampling was done in times when the patients, e.g., had a cold, covid, any other viral infection(s), or some other illness that could have influenced this measurement. Is it possible somehow to veryfy this in the databases, e.g., what ICD10 codes were associated with the baseline visit (if recorded at all)?

6. There are a few typos here and there. A thorough read is suggested.

+1. This is only a theoretical question for the Authors. From the supplementary data it can be read that for most cancer types the OR was over 1, meaning that the higher values are associated with higher cancer risk. Is it possible to investigate the raw data, instead of low/normal and high groups? One can assume, that if raw values are included in a statistical model, maybe some kind of J- or U-shaped risk would appear as a result. What are Authors' thoughts on this question?

**Reviewer**	**2**
**Name**	**Klawonn, Frank**
**Affiliation**	**Ostfalia Hochschule fur angewandte Wissenschaften**
**Date**	**24-Apr-2026**
**COI**	**none**

The statistical approach is in principle correct. It is not clear why age had been divided into discrete groups instead of using age directly as a continuous variable.

The smoking status (ever or never) is quite vague. How is "ever" defined? Does it count if one had once tried a cigarette? Considering smoking behaviour in such general manner might not provide reliable insight w.r.t. the influence of smoking.

**Reviewer**	**3**
**Name**	**Oke, Jason**
**Affiliation**	**University of Oxford, Nuffiled Department of Primary Care**
**Health Sciences**	
**Date**	**09-Jul-2026**
**COI**	**none**

This is well-written, the principal findings are clearly stated and the limitations are thoughtfully considered. I have a few comments/questions.

1.Cohort entry definition. It isn't completely clear what defines cohort entry. Is the index date the date of the first eligible platelet or CRP test, and so did all follow-up start from this date?

2. It isn't obvious whether there are one combined cohort, two overlapping cohorts, or separate platelet and CRP cohorts. Were patients with both tests included in both analyses? This just needs clarifying.

3. It's not stated when BMI, smoking status and multimorbidity were measured. There is no explanation of what happened with missing covariates. Is this a complete-case analysis or was imputation used?

4. The word "incidence" (rate) is used repeatedly but what is being estimated is 1-year risk. As cancer is relatively rare, the risk and the incidence rate will probably coincide. What might be important however, is whether the time at which the diagnoses occur changes things. Would it be possible to check (with one example) that no material differences are observed using a method such as Cox that explicitly takes into account the time of diagnosis.

5. Ethnicity was in CPRD and HES which source took precedence if they disagreed?

6. CPRD studies almost always include CPRD Independent Scientific Advisory Committee approval (or equivalent), ethics approval, protocol number. Can these details be added?

7. Why was the DOR used? It is not an easy metric to interpret in my opinion. A risk ratio would be easier to interpret. Perhaps provide a justification and an explanation of how to interpret the DOR.

A PPV would be more useful in interpreting this in terms of guidelines. Also, use of the term predictive value is used in the first sentence in the discussion suggests a PPV to me. Consistent terminology?

8. The final sentence emphasises the importance of ethnically diverse cohorts, but I think it would be better to reiterate the main findings. — that thrombocytosis and raised CRP appeared to have broadly consistent predictive value across ethnic groups in UK primary care.

9. Check for repetition in the discussion. The discussion is somewhat repetitive with the opening two paragraphs make essentially the same point—that there was little evidence of ethnic variation overall.

10. The discussion refers to statistically significant interaction terms (e.g. colorectal, renal, leukaemia), but there is relatively little discussion of the magnitude or clinical importance of these differences. Readers would benefit from more emphasis on effect sizes and their potential clinical relevance rather than p-values alone.

11. “The overarching strength of this study was the unprecedented size” "Very large" or "one of the largest cohorts to date" seems more appropriate.

thanks.

VERSION 1 - AUTHOR RESPONSE

Reviewers’ comments	Author response
**Reviewer 1: Dr. Zoltan Herold, Semmelweis University**	
1. It is suggested to include some extra information in the Introduction.<br><br>a) Specifically it was not detailed how platelet and CRP changes with age. There are a few articles about this topic.<br><br>b) Moreover, it is worth to mention how cancer develops, specifically the timeframe, why is it interesting to investigate it in this situation.<br><br>c) Furthermore, maybe the Authors can mention some of previous works that tried to investigate how platelet and the varios calculated biomarkers (such as the "personalized	a) Platelet count and CRP levels are influenced by a range of factors, including age, gender BMI, comorbidity, smoking status, and socioeconomic deprivation. As the focus of this manuscript is the relationship between ethnicity, platelet count and CRP levels, and subsequent cancer incidence, rather than the determinants of platelet count or CRP levels themselves, we felt a detailed discussion of the factors influencing these biomarkers was beyond the scope of the introduction and may detract from the main rationale for the study. However, age is a very important consideration and potential confounding factor so we have adjusted or stratified by age throughout the analyses. We have also adjusted for gender, BMI, comorbidity, socioeconomic deprivation, smoking status, along with age, in the modelling analyses.<br><br>b) We have added further rationale regarding why routinely measured blood tests may provide opportunities for earlier cancer detection. Specifically, we have added the following text to the Introduction (lines 84-86):<br><br>***“As cancers develop, they can induce systemic inflammation and alter haematological and inflammatory markers, sometimes before symptoms arise, creating opportunities for earlier detection through routinely requested blood tests. For example…”***<br><br>We have also added further clarification regarding the selection of the one-year timeframe and cited relevant literature exploring changes in blood tests prior to cancer diagnosis (please see response to comment 3).<br><br>c) We have described the previous work (that was led by our team) that investigated how thrombocytosis has high

indicator thrombocytosis", PLR, SII, PIV, etc.) are associated with cancer incidence.	PPVs for cancer incidence in the introduction (lines 87-90). To the best of our knowledge, other biomarkers such as personalised indicator thrombocytosis, PLR, SII, PIV and others have been investigated in relation to cancer prognosis or treatment response rather than cancer diagnosis / incidence. We have conducted an additional quick search of the literature and cannot find studies evaluating the association of cancer diagnosis or incidence with these biomarkers. However, if we have missed these, we would be very interested to read them and discuss them in this manuscript if the reviewer is happy to signpost them to us.
2. In Methods, some additinal details should be included, such as how the various cancer types were recorded in the database (ICD10 codes) and how Authors used these?	We have added the following text to the methods (lines 123-133.<br><br>*"Cancer diagnoses were identified from the cancer registry **using ICD-10 codes (Supplementary materials 1)**".*<br><br>The additional Supplementary materials 1 lists the ICD-10 codes used to identify cancer diagnoses, and the site to which they were assigned.
3. In most sources the development time of malignant tumors is usually between a 3-5 years range, depending on the source. Authors should include some citation, or give a bit more details on why they chose only to investigate cancers developing within a year timeframw following labor sampling.	Lines 136-138 in the methods explain the one-year time frame with provided citations, however we acknowledge that this could be further justified and have added the following text to lines 138-139, with additional citations.<br>***"and to capture the pre-diagnostic rise in haematological indices before cancer diagnosis (26, 27)"***
4. In the general cohort statitistics it is advised to add an additional (supplementary) table to give details on the cancer details, including the number of cancers developed in total, in the various cancer types (crc, breast, etc.), TNM stages, etc. If these daata were previously	Thank you for this excellent suggestion. We have added this table into an additional a supplementary file (7) and have added the following text to the results, lines (262-264).<br><br>***"A total of 91,585 (1.9%) patients were diagnosed with cancer in the year following their platelet count test, and 22,089 (2.7%) in the year after their CRP test. Supplementary materials 7 presents cancer site and stage at diagnosis."***

published, a citation would also work	
5. In the case of CRP data, it is a bit strange that the percentage of patients with high CRP is between 20-40%. This suggests that the sampling was done in times when the patients, e.g., had a cold, covid, any other viral infection(s), or some other illness that could have influenced this measurement. Is it possible somehow to veryfy this in the databases, e.g., what ICD10 codes were associated with the baseline visit (if recorded at all)?	CRP testing in UK primary care is often undertaken in the context of suspected inflammation or infection which contributes to the higher proportion of patients with an elevated CRP compared to the general population. In contrast, platelet count is measured as part of a full blood count and is requested more broadly, rather than in response to a specific clinical indication, contributing to the relatively lower prevalence of thrombocytosis in the platelet cohort.<br><br>We have added the following paragraph to the discussion acknowledging this (lines 386-392).<br><br>***"The relatively higher proportion of patients presenting with a raised CRP level (≥20%, Figure 2) compared to those with thrombocytosis (≥2%, Figure 1) likely reflects primary care testing practices. CRP testing is often undertaken in the context of suspected inflammation or infection, often supporting clinical decision making regarding antibiotic prescribing. In contrast, platelet count is measured as part of a full blood count, which is requested across a broader range of clinical contexts, with thrombocytosis often occurring as an incidental finding."***<br><br>We have also expanded this section to return to the primary focus of our manuscript, which ties this paragraph in nicely to the next.<br><br>***"However, the reasons underlying the differences by ethnicity are less clear. The median platelet counts presented in Supplementary materials 4 indicated that Black patients have lower counts than their White and Asian counterparts across all age groups, raising an important concern regarding the use of universal thrombocytosis thresholds in cancer risk estimation. If baseline platelet distributions differ by ethnicity, a similar absolute increase in platelet count may not result in the same likelihood of exceeding current thrombocytosis thresholds, potentially affecting who is identified for further investigation in primary care."***
6. There are a few typos here and there. A thorough read is suggested.	We have thoroughly proof read the manuscript and corrected typos.

<table>
<tr><td>+1. This is only a theoretical question for the Authors. From the supplementary data it can be read that for most cancer types the OR was over 1, meaning that the higher values are associated with higher cancer risk. Is it possible to investigate the raw data, instead of low/normal and high groups? One can assume, that if raw values are included in a statistical model, maybe some kind of J- or U-shaped risk would appear as a result. What are Authors' thoughts on this question?</td><td>Thank you for this interesting suggestion. We agree that modelling platelet count and CRP as continuous variables may provide additional insights into the relationship between these markers and cancer risk. However, this represents a different research question from that addressed in the current study. Our primary aim was to investigate whether the association between clinically relevant platelet and CRP thresholds and subsequent one-year cancer incidence differs by ethnicity. We therefore used established thresholds for thrombocytosis and raised CRP, as these are supported by previous research, align with current NICE guidance, and are directly applicable to clinical decision-making in primary care.

Modelling continuous values, allowing for potential non-linear relationships while simultaneously investigating interactions with ethnicity, would substantially increase the complexity of the analyses, and importantly, the interpretation. This is beyond the scope of the present manuscript, however, we agree that investigating the underlying shape of these associations is an interesting area for future research. Our group continues to explore the relationship between routinely collected blood test values and cancer risk in ongoing work.</td></tr>
<tr><td colspan="2">Reviewer 2: Prof. Frank Klawonn, Ostfalia Hochschule fur angewandte Wissenschaften</td></tr>
<tr><td>The statistical approach is in principle correct.
a) It is not clear why age had been divided into discrete groups instead of using age directly as a continuous variable.
b) The smoking status (ever or never) is quite vague. How is "ever" defined? Does it count if one had once tried a cigarette? Considering smoking</td><td>a) Including indicator variables for age groups (excluding one category as the reference) in models is a very common approach in the field. It has the great advantage of allowing the exploration of different trends across groups rather than being constrained to a linear relationship, as would be the case if adding age as a continuous variable. One could use e.g. splines or fractional polynomials to better account for non-linear relationships with age. However, that is adding unnecessary complexity as the effects of age on cancer risk are not the focus in this manuscript, being very well known already. It is included in the model as an adjustment for confounding only.

b) Patients were classified as being an ever-smoker if they had a smoking related medical code or smoking cessation prescription code in their primary care record. Smoking status was categorised as ever-or never- over classification into current-smokers, past-smokers, and never-smokers for a number of reasons. Firstly, the</td></tr>
</table>

behaviour in such general manner might not provide reliable insight w.r.t. the influence of smoking.	recording of smoking status in English primary care is variable (doi:10.1093/fampra/cmq046) and longitudinal updating of smoking behaviour remains inconsistent (doi:10.1186/s12911-016-0400-6). The medical codes used to describe smoking status were often ambiguous in terms of smoking intensity, e.g. 'smoking cessation attempts'. Additionally, between half to two-thirds of past-smokers are able to stop smoking without GP or pharmacotherapy assistance (doi:10.1136/bmjopen-2014-007301), meaning smoking cessation may not be captured within the primary care record. As a result, individuals recorded as current smokers may have in practice have stopped smoking, while former smokers may have relapsed. Categorisation into ever- versus never- smokers was therefore considered a more robust approach that reduced the risk of misclassification, albeit at the expense of reduced granularity regarding smoking intensity, duration, and current status, given the dataset used.
**Reviewer 3 : Dr. Jason Oke, University of Oxford**	
This is well-written, the principal findings are clearly stated and the limitations are thoughtfully considered. I have a few comments/questions.	
1.Cohort entry definition. It isn't completely clear what defines cohort entry. Is the index date the date of the first eligible platelet or CRP test, and so did all follow-up start from this date?	Yes, this is correct. We have made the following amendments to the methods (lines 125-127) to clarify – ***"Cohort entry (the index date) was defined as the date of the first eligible platelet or CRP test during the study period, from which follow-up commenced."***
2. It isn't obvious whether there are one combined cohort, two overlapping cohorts, or separate platelet and CRP cohorts. Were patients with both tests included in both analyses? This just needs clarifying.	They are separate platelet and CRP cohorts, though many patients appeared in both datasets. We have added the following text to the methods (lines 127 – 130) to clarify - ***"Separate analyses were conducted for platelet count and CRP. Accordingly, two cohorts were assembled: one based on the first eligible platelet test and one based on the first eligible CRP test. Patients with both tests could contribute to both cohorts, with cohort entry defined independently for each analysis."***

3. It's not stated when BMI, smoking status and multimorbidity were measured. There is no explanation of what happened with missing covariates. Is this a complete-case analysis or was imputation used?	We have clarified in the Methods (lines 171-178) when covariates were measured and how missing data were handled.<br>***"Smoking status, BMI, and multimorbidity were all ascertained from records available prior to cohort entry (the index test date). BMI was defined using the most recent recorded measurement before the index date. Patients without a recorded smoking status were classified as never smokers, while those without recorded conditions contributing to the Cambridge Multimorbidity Score were assigned a score of 0 "no identified conditions". Patients without a recorded BMI were retained in the analysis as a separate 'BMI not recorded' category. Multiple imputation was not undertaken because the absence of a BMI measurement in routinely collected primary care data is unlikely to be missing at random."***
4. The word "incidence" (rate) is used repeatedly but what is being estimated is 1-year risk. As cancer is relatively rare, the risk and the incidence rate will probably coincide. What might be important however, is whether the time at which the diagnoses occur changes things. Would it be possible to check (with one example) that no material differences are observed using a method such as Cox that explicitly takes into account the time of diagnosis.	Thank you for this comment. We recognise that the terminology 'incident rate' is misleading and have amended terminology throughout the manuscript to 'one-year cancer incidence', maintaining consistency with similar work in cancer epidemiology.<br><br>Time to event analyses is outside the scope of the present manuscript but is something our team is exploring with ongoing work.
5. Ethnicity was in CPRD and HES which source took precedence if they disagreed?	CPRD took precedence over HES, following a previously published algorithm designed for this programme of work. We have added the following text with citation to signpost to this.<br>***"Ethnicity was primarily obtained from CPRD and extracted from HES APC when missing from CPRD, following a previously published algorithm (21)"***
6. CPRD studies almost always include CPRD	Yes - we have added this information to lines 119-120.

Independent Scientific Advisory Committee approval (or equivalent), ethics approval, protocol number. Can these details be added?	***"The Electronic Research Applications Portal granted ethical approval for this study, reference number 21_000315*.*"***
7. Why was the DOR used? It is not an easy metric to interpret in my opinion. A risk ratio would be easier to interpret. Perhaps provide a justification and an explanation of how to interpret the DOR. A PPV would be more useful in interpreting this in terms of guidelines. Also, use of the term predictive value is used in the first sentence in the discussion suggests a PPV to me. Consistent terminology?	Thank you for this helpful comment. We have revised the manuscript to refer to odds ratios (ORs) throughout, rather than diagnostic odds ratios (dORs), to avoid confusion.<br>As our primary aim was to investigate whether the association between raised platelet count and CRP and subsequent one-year cancer incidence differed by ethnicity after adjustment for potential confounders, multivariable logistic regression was the most appropriate analytical approach. The resulting ORs provide an adjusted measure of the relative association between blood test result and cancer across ethnic groups. Although PPVs are clinically useful for informing referral thresholds and guidelines, estimating PPVs was not the objective of this study. PPVs are influenced by the underlying prevalence of disease and healthcare utilisation, including differences in testing practices and access to care, which may differ between ethnic groups.<br><br>We have amended the first sentence of the discussion to: ***"This large UK primary care study found the association between thrombocytosis or a raised CRP result for one-year cancer incidence was generally consistent across ethnic groups"***
8. The final sentence emphasises the importance of ethnically diverse cohorts, but I think it would be better to reiterate the main findings. — that thrombocytosis and raised CRP appeared to have broadly consistent predictive value across ethnic groups in UK primary care.	We agree. We have amended the final sentence to *"Overall, these findings* ***provide reassurance that thrombocytosis and raised CRP appear to have broadly consistent associations with one-year cancer incidence across ethnic groups in UK primary care****."*.<br><br>We have also amended the abstract conclusion to ***"This large primary care study provides reassurance that thrombocytosis and raised CRP appear to have broadly consistent associations with one-year cancer incidence across ethnic groups in UK primary care."***
9. Check for repetition in the discussion. The discussion is somewhat	Thank you for flagging this. We have removed the second sentence of the discussion and have combined the first sentence with the second paragraph to reduce repetition.

repetitive with the opening two paragraphs make essentially the same point—that there was little evidence of ethnic variation overall.	
10. The discussion refers to statistically significant interaction terms (e.g. colorectal, renal, leukaemia), but there is relatively little discussion of the magnitude or clinical importance of these differences. Readers would benefit from more emphasis on effect sizes and their potential clinical relevance rather than p-values alone.	We have added the effect sizes to the main text (results, lines 329 – 330; discussion, lines 376-377). We are cautious to interpret the clinical importance of these findings due to the number of statistical comparisons performed which increase the likelihood of chance associations which we discuss in the discussion, lines 378-385.
11. "The overarching strength of this study was the unprecedented size" "Very large" or "one of the largest cohorts to date" seems more appropriate	Agreed - we have amended "unprecedented" to "very large".

VERSION 2 - REVIEW

**Reviewer**	**1**
**Name**	**Herold, Zoltan**
**Affiliation**	**Semmelweis University, Department of Clinical Oncology**
**Date**	**23-Sep-2026**
**COI**	

Authors addressed all my concerns, I have no further question.

I recommend the

**Reviewer**	**2**

Name Klawonn, Frank

Affiliation Ostfalia Hochschule fur angewandte Wissenschaften

Date 09-Oct-2026

COI

The authors have answered my questions properly.
