# Supplementary material for: Cancer incidence following platelet count and C-reactive protein testing across ethnic groups in English primary care: a cohort study of 5 million patients
Source: BMJ Open. 2026 Oct 26;16(10):e120128. doi: 10.1136/bmjopen-2026-120128 (PMC13711558; doi:10.1136/bmjopen-2026-120128)
Supplement: Supplementary file 2 [file bmjopen-16-10-s001.docx]

| **Cancer site** | **ICD-10 code(s)** |
| --- | --- |
| In-situ tumours | D* |
| Lip, oral cavity and pharynx | C00–C14 |
| Oesophagus | C15 |
| Stomach | C16 |
| Small intestine | C17 |
| Colon | C18 |
| Rectum | C19–C20 |
| Anus | C21 |
| Pancreas | C25 |
| Head and neck (excluding oral cavity/pharynx and brain) | C30–C32, C73, C76.0* |
| Larynx | C32 |
| Lung | C34 |
| Melanoma | C43 |
| Non-melanoma skin cancer | C44 |
| Mesothelioma | C45 |
| Soft tissue sarcoma | C49 |
| Breast | C50 |
| Cervix | C53 |
| Uterus | C54–C55 |
| Ovary | C56 |
| Prostate | C61 |
| Testis | C62 |
| Kidney | C64 |
| Bladder | C67 |
| Brain | C71 |
| Thyroid | C73 |
| Unknown primary | C77–C80 |
| Hodgkin lymphoma | C81 |
| Non-Hodgkin lymphoma | C82–C85 |
| Myeloma | C90 |
| Leukaemia | C91–C95 |
|  |  |

**Derived groupings used in the analyses**

| **Group** | **ICD-10 code(s)** |
| --- | --- |
| Oesophago-gastric cancer | C15–C16 |
| Colorectal cancer | C18–C20 |
