# Supplementary material for: Cancer incidence following platelet count and C-reactive protein testing across ethnic groups in English primary care: a cohort study of 5 million patients
Source: BMJ Open. 2026 Oct 26;16(10):e120128. doi: 10.1136/bmjopen-2026-120128 (PMC13711558; doi:10.1136/bmjopen-2026-120128)
Supplement: Supplementary file 8 [file bmjopen-16-10-s007.docx]

Supplementary Materials 7. Patients diagnosed with cancer in the year following their platelet or CRP test, by cancer site and stage at diagnosis.

| **Platelet cohort** | | | | |
| --- | --- | --- | --- | --- |
| **Cancer site** | **n** | **Early stage (I-II)** | **Advanced stage (III-IV)** | **Unknown stage** |
| Bladder | 2,851 | 1,062 | 443 | 1,346 |
| Brain | 1,258 | 0 | 3 | 1,255 |
| Breast | 8,484 | 4,888 | 1,142 | 2,454 |
| Cervix | 557 | 306 | 159 | 92 |
| Colorectal | 13,135 | 4,474 | 6,546 | 2,115 |
| Kidney | 2,788 | 719 | 966 | 1,103 |
| Leukaemia | 2,892 | 4 | 12 | 2,876 |
| Lung | 11,522 | 1,549 | 7,675 | 2,298 |
| Lymphoma | 4,483 | 684 | 1,706 | 2,093 |
| Melanoma | 2,087 | 1,606 | 180 | 301 |
| Myeloma | 1,925 | 128 | 79 | 1,718 |
| Oesophagus | 2,584 | 319 | 1,192 | 1,073 |
| Oral | 1,920 | 260 | 873 | 787 |
| Other | 4,194 | 412 | 1,071 | 2,711 |
| Ovary | 2,292 | 643 | 1,079 | 570 |
| Pancreas | 3,344 | 255 | 1,519 | 1,570 |
| Prostate | 14,699 | 4,758 | 4,682 | 5,259 |
| Stomach | 2,255 | 277 | 935 | 1,043 |
| Testis | 235 | 119 | 24 | 92 |
| Uterus | 2,000 | 1,311 | 360 | 329 |
| Small Intestine | 404 | 30 | 190 | 184 |
| Anus | 311 | 65 | 101 | 145 |
| Mesothelioma | 753 | 36 | 182 | 535 |
| Soft tissue sarcoma | 367 | 45 | 72 | 250 |
| Head and neck | 1,355 | 301 | 308 | 746 |
| Unknown primary | 2,890 | 1 | 389 | 2,500 |
| **Total** | **91,585 (100%)** | **24,252 (26.5%)** | **31,888 (34.8%)** | **35,455 (38.7%)** |
| **CRP cohort** | | | | |
| **Cancer site** | **n** | **Early stage (I-II)** | **Advanced stage (III-IV)** | **Unknown stage** |
| Bladder | 588 | 258 | 110 | 220 |
| Brain | 279 | 1 | 1 | 277 |
| Breast | 1,594 | 983 | 248 | 363 |
| Cervix | 90 | 43 | 23 | 24 |
| Colorectal | 3,385 | 1,229 | 1,734 | 422 |
| Kidney | 716 | 202 | 289 | 225 |
| Leukaemia | 699 | 2 | 1 | 696 |
| Lung | 3,247 | 565 | 2,202 | 480 |
| Lymphoma | 1,417 | 259 | 641 | 517 |
| Melanoma | 396 | 321 | 35 | 40 |
| Myeloma | 611 | 56 | 36 | 519 |
| Oesophagus | 579 | 102 | 305 | 172 |
| Oral | 444 | 99 | 261 | 84 |
| Other | 1,180 | 163 | 319 | 698 |
| Ovary | 584 | 133 | 303 | 148 |
| Pancreas | 1,095 | 136 | 566 | 393 |
| Prostate | 2,695 | 989 | 1,092 | 614 |
| Stomach | 559 | 133 | 263 | 163 |
| Testis | 42 | 19 | 6 | 17 |
| Uterus | 349 | 236 | 69 | 44 |
| Small Intestine | 127 | 24 | 67 | 36 |
| Anus | 70 | 21 | 30 | 19 |
| Mesothelioma | 197 | 22 | 57 | 118 |
| Soft tissue sarcoma | 80 | 25 | 20 | 35 |
| Head and neck | 275 | 73 | 90 | 112 |
| Unknown primary | 791 | 5 | 107 | 679 |
| **Total** | **22,089 (100%)** | **6,099**  **(27.6%)** | **8,875**  **(40.2%)** | **7,115**  **(32.2%)** |
